# Inducing Craving for Alcohol in the Laboratory

**Published:** 1999

**Authors:** Mark D. Litt, Ned L. Cooney

**Affiliations:** Mark D. Litt, Ph.D., is an associate professor in the Department of Behavioral Sciences and Community Health at the University of Connecticut Health Center, Farmington, Connecticut. Ned L. Cooney is at the Veterans Affairs Connecticut Healthcare System, West Haven, Connecticut

**Keywords:** AOD (alcohol and other drug) craving, alcohol cue, relapse prevention, laboratory measurement, classical conditioning, treatment model, prevention research, AOD use behavior, empirical study, expectancy, visual perception, psychological AODC (causes of alcohol and other drug use), biological AODC, emotion, laboratory study, literature review, context dynamics

## Abstract

Research on the mechanisms of craving often involves inducing craving in subjects in controlled settings. This article describes techniques that have been used to induce craving for alcohol, including (1) exposing subjects to actual alcoholic beverages, (2) exposing subjects to visual representations of alcoholic beverages, (3) manipulating the subjects’ mood states, and (4) controlling environmental settings. The intensity of craving can be rated by the subjects themselves or can be assessed by clinicians through behavioral observations or the measurement of certain physiological responses. Success in inducing craving in the laboratory, however, has been inconsistent. Ultimately, researchers may need to monitor subjects’ craving responses in actual environmental settings.

Many researchers and clinicians consider craving for alcohol a precursor to relapse among alcoholics in treatment (Marlatt and Gordon 1985). The role of craving in relapse is controversial, however, and research on the subject is hampered by the lack of a generally accepted definition of craving. Rankin and colleagues (1979) defined craving as a “central state,” synonymous with a desire or disposition to drink alcohol. Using this approach, craving can be considered a motivational state (i.e., a condition that increases the probability of seeking and consuming alcohol or other drugs [AODs]). This does not imply, however, that craving always leads to drinking. Although this definition lacks precision to some extent, it has the advantage of differentiating craving from intention, expectancies, or automatic behaviors (i.e., “habit”) (see the article in this issue by Rohsenow and Monti, pp. 225–232).

Craving may be triggered by exposure to an object, environment, or emotion that a person has come to associate with alcohol consumption. Such stimuli are called alcohol-related cues (ARCs). Ludwig and colleagues (1974) suggested that the ability of ARCs to elicit craving may be acquired through a learning process called classical conditioning (see sidebar by Tiffany, p. 216). This form of learning occurs when a stimulus that would not normally elicit any particular response (i.e., a neutral stimulus) is repeatedly associated, or paired, with a stimulus that does elicit a specific response. The previously neutral stimulus is referred to as a cue. Thus, exposure to ARCs may eventually elicit mental, behavioral, and physiological reactions similar to those evoked by the actual consumption of alcohol or by withdrawal.^1^ Such cues may include the sight or smell of an alcoholic beverage; the familiar surroundings of a favorite bar; or the onset of an emotional state, such as anger or depression, that a person is accustomed to reacting to by drinking.

Some researchers have explored the possibility of diminishing craving by repeatedly exposing subjects to ARCs without permitting them to drink (Laberg 1990). Proponents of this approach purport that the procedure can diminish and eventually abolish craving by weakening the association between cue and response.

Research on the mechanisms of craving is required to improve our understanding of the development of alcoholism and to support treatment and prevention efforts. Therefore, researchers have developed techniques to induce craving for alcohol in controlled settings. This article briefly reviews techniques for measuring craving and describes approaches to inducing craving that use the following strategies: (1) exposing subjects to actual alcoholic beverages, (2) exposing subjects to visual representations of alcoholic beverages, (3) manipulating subjects’ mood states, and (4) controlling environmental settings.

## Measuring Craving

Craving can be measured directly by asking subjects to rate the strength of their urge to drink or indirectly by observing subjects’ behavior in response to ARCs or to the consumption of alcohol itself. Craving can also be assessed indirectly by measuring certain physiological responses that appear to accompany craving, such as increased salivation and swallowing. Other measurable physiological responses include changes in heart rate and in the electrical properties of the skin surface. (See the article in this issue by Drobes and Thomas, pp. 179–186, for a review of the applications, advantages, and disadvantages of techniques for measuring craving.)

## Exposure to Alcoholic Beverages

The first attempts to elicit craving in the laboratory involved the presentation of those cues considered most likely to evoke an appropriate response (i.e., the most salient cues) (Laberg 1990). In the most basic application of this approach, the experimenter places a standard alcoholic drink on a table in front of the subject and instructs the subject to look at the beverage, hold it (at arm’s length in some studies), and sniff it repeatedly. To minimize the possibility of provoking relapse, researchers usually instruct alcohol-dependent subjects not to drink the beverage and provide them with no opportunity to consume it.

The effectiveness of this frequently used method for eliciting craving is variable. Although researchers using this approach have found overall increases in self-reported desire to drink (Laberg 1990), other researchers have noted that reactivity *rates* are only about 50 to 60 percent (i.e., only approximately 50 to 60 percent of subjects report increased craving [Litt et al. 1990]). This procedure can be rendered more effective by offering the subject his or her choice of alcoholic beverage (Laberg 1990) and by mixing and pouring the drink (or allowing the subject to do so) in the manner to which the subject is accustomed.^2^

Pomerleau and colleagues (1983) combined olfactory and visual cues in a series of 5-minute “sniff” trials involving 8 alcoholics in treatment, 10 nonalcoholics, and 2 recovering alcoholics. As a basis for comparison, the subjects were first instructed to sniff cedar chips to determine their reaction to a distinctive but nonalcoholic odor. Each subject subsequently sniffed his or her favorite alcoholic beverage from an open container bearing the manufacturer’s label. Aggregate reactivity to alcohol, measured as swallowing and as self-reported craving, was higher among alcohol-dependent subjects than among nonalcoholic subjects. Of the 10 alcoholic participants in this study, 9 showed significantly increased salivation after sniffing alcohol, a reactivity rate of 90 percent. However, only 5 of the 10 alcoholic participants reported subjective increases in craving.

Stormark and colleagues (1995) evaluated physiological responses and self-reported craving among alcoholics in treatment and among social drinkers^3^ exposed to olfactory stimuli. Alcoholic cues included beer and vodka; neutral cues included vanilla and soap. Based on the results of prior testing, these stimuli were further distinguished by the ease with which they could be identified by smell. Neither neutral odors nor the low-potency (i.e., less identifiable)^4^ alcohol odor (i.e., vodka) elicited significant differences in physiological reactivity or self-reported craving. However, alcoholic participants showed decreased heart rates in response to the high-potency alcohol odor (i.e., beer). This reaction is considered a physiological indicator of the orienting response, in which attention is directed to a novel and potentially important stimulus that may require a response. Alcoholic subjects also reported increased difficulty in their perceived ability to refuse a drink in response to high-potency alcohol odors than did social drinkers. These reactions in the alcoholic subjects are consistent with what would be considered a craving response.

The most successful means of eliciting craving in the laboratory is to present a subject with alcohol and allow him or her to consume it. Although the subject may be urged to resist drinking if possible (Cooney et al. 1997), the subject must be informed that he or she will not be prevented from drinking. In a series of experimental sessions conducted over a period of 4 months, Laberg (1990) compared the effects on alcoholic subjects of an initial (i.e., priming) dose of an alcoholic beverage (i.e., vodka) or a “soft drink” (i.e., a Tom Collins mix without alcohol). Experimenters measured each subject’s self-reported cravings as well as his or her behavioral and physiological responses three times during the study: (1) when the subject was told he or she would receive alcohol, (2) when the subject consumed the priming dose, and (3) when the subject was offered two extra drinks 45 minutes after consuming the priming dose (i.e., the drinking test).^5^ At each session, one-half of the subjects who had been told that they were to receive alcohol were actually given water with one drop of vodka on the rim of the bottle. Similarly, one-half of the subjects who had been told they were to receive a nonalcoholic drink were given alcohol.

In these studies, the rates of craving reactivity among alcoholics were high. Interestingly, the self-reports of alcohol craving by alcoholic subjects were influenced more by whether a subject believed that the priming drink was alcoholic or nonalcoholic than by the drink’s actual contents. These studies suggest that the promise, or expectancy, of a future alcoholic drink is a potent cue for craving, especially when a drink has already been consumed (see also DeWit 1996).

Although the combination of priming and expectancy induces craving responses, the approach presents conceptual difficulties, both as an experimental procedure and as a potential component of treatment programs. Conceptually, craving poses the greatest threat to recovering alcoholics who are currently abstinent. Allowing subjects a priming dose of alcohol in the laboratory provides no information on factors that influence consumption of a person’s *first* drink; rather, these procedures explore the effects of craving on further alcohol consumption after drinking is initiated. Some clinicians have suggested that offering alcohol to a person who has been trying to abstain might counteract the effects of therapy. However, many researchers have safely administered alcohol in experimental settings to selected, well-supervised subjects who have been informed of the risks and benefits involved, as in any other type of medical research (Modell et al. 1993).

Yet the presentation of alcohol cues alone, even of a favored beverage mixed in front of a subject, has not always proven to be a salient stimulus. Additionally, if laboratory craving is to be considered an analog for craving in the natural environment, then craving reactivity should predict drinking behavior outside the laboratory. Reactivity to alcohol cues has predicted relapse in some studies but not in others (see Cooney et al. 1997). Evidence indicates that alcohol cues alone may not be sufficiently salient to elicit craving from many alcoholics in the absence of an opportunity to consume.

## Exposure to Alcohol-Related Visual Images

Because visual representations of alcohol are ubiquitous in our society (e.g., in advertisements and storefronts), it is reasonable to examine reactivity in response to the presentation of purely visual stimuli. Although visual stimuli have been used to elicit reactivity in drug abusers, such cues have rarely been employed to study alcoholism. In one study (Eriksen and Götestam 1984), alcoholic subjects who had viewed slides of alcohol-related subject matter (e.g., liquor stores, alcoholic beverage containers, and scenes of people consuming alcoholic beverages) reported significantly greater increases in subjective alcohol craving and anxiety levels than did alcoholic subjects who had viewed slides of non-alcohol-related subject matter (e.g., a row of storefronts or scenes of people drinking milk, tea, or coffee). Physiological measures did not differ between groups of subjects viewing the two sets of slides. No data relating reactivity to later drinking were reported.

Cassisi and colleagues (1998) measured the physiological responses of nonalcoholic social drinkers who viewed slides depicting alcohol- and non-alcohol-related beverage advertisements from popular magazines. Lighter drinkers (i.e., drinkers who consumed an average of two alcoholic beverages per week) tended overall to show significant decreases in heart rate in response to both types of slides, an orienting response to incoming information regardless of its content. Heavier drinkers (i.e., drinkers who consumed 16 or more alcoholic beverages per day) overall showed no change in heart rate but demonstrated increased skin conductance, indicating activation of the nervous system characteristic of anxiety. This finding is consistent with decisionmaking-focused theories, postulating that urges reflect a conflict between “motivation to use” and “intention to stay abstinent” (see the article in this issue by Breiner and colleagues, pp. 197–206).

In general the few studies that have relied solely on visual cues to elicit craving have produced conflicting findings, and no study has linked visual cue reactivity to subsequent drinking behavior. Thus, visual cues alone are probably not sufficiently salient for research purposes.

## Alteration of Mood State

Much of the research on cue reactivity in alcoholics has focused on external cues, primarily the sight or smell of alcoholic beverages. It has become increasingly apparent that internal cues, such as mood states, can also elicit the desire for alcohol if, for example, a person habitually drinks alcohol when in a particular mood (Cooney et al. 1997). Alcoholics have often reported that their relapses occurred during negative mood states, such as depression, anger, or interpersonal stress (Marlatt and Gordon 1985). In addition, depression and anger have been shown to trigger opiate craving as well as symptoms similar to those of withdrawal in abstinent opiate abusers (Childress et al. 1993).

Occasionally, videotapes have been used in studies of alcohol craving to induce desired moods. This type of research is typically conducted by showing subjects film excerpts that have been independently rated as inducing specific mood states—for example, joy (e.g., *The Sound of Music*), disgust (e.g., *Dawn of the Dead*), or anger (Kornreich et al. 1998). Cooney and colleagues (1997) presented a brief review of the way in which the manipulation of mood states is used in alcoholism research. The basic procedure often involves interviewing the participant about his or her recent drinking history (e.g., a relapse), with an emphasis on eliciting information about the person’s mood states during these episodes. The participants are asked to relate the experience in as much detail as possible. A so-called guided imagery script is then prepared incorporating the details from that event, including strong and frequent inducements to cause the person to experience the same mood as in the original incident. This script is usually read to the person while he or she is in a relaxed but attentive state, before or during the presentation of a preferred alcoholic beverage or exposure to an ARC. Mood ratings, as well as craving ratings, are taken to ensure that the appropriate mood is induced.

In an initial trial of mood induction to elicit craving, Litt and colleagues (1990) employed scripts in which mood state alone was suggested without including any mention of use or presence of alcohol or alcohol cues. Both positive and negative moods were induced using a hypnotic induction procedure. Results indicated that negative mood alone was sufficient to elicit the desire to drink in five of eight of the subjects but that alcoholic beverage presentation alone did not elicit craving. Relaxation accompanying hypnotic induction may have been sufficient to dampen the subjects’ responses to alcohol cues in the absence of the arousing mood states that tend to stimulate alcohol-seeking behavior (see the articles in this issue by Rohsenow and Monti, pp. 225–232, and Breiner and colleagues, pp. 197–206). Thus, procedures that elicit deep relaxation may not be optimal for eliciting high rates of craving.

More recently, investigators have administered to subjects guided imagery scripts that essentially recreate relapse episodes, complete with whatever beverage cues the subjects encountered during their original episodes. The most effective mood states for inducing craving are consistently negative ones, often involving high arousal, as in anger or anxiety (Cooney et al. 1997). The use of such high-emotion, high-risk scripts has produced relatively high reactivity rates in subjects, which in turn have sometimes been shown to predict relapse. To date, guided imagery procedures have proven to be the most effective and efficient means to elicit reports of craving, although more effective means may be available (Cooney et al. 1997).

## Manipulating the Setting

Laboratory studies of craving have often been criticized because of the artificial nature of the drinking environment. Thus, subjects consuming alcohol in the controlled setting of a hospital or laboratory are less likely to experience the “loss of control” that may occur when they drink in their homes or in their favorite bars (Sobell et al. 1972; Ludwig et al. 1977). Studies in which drinking is not allowed provide an even more artificial environment for inducing craving. Such studies, which are often located in a treatment setting, may actually inhibit craving (or at least *reported* craving) in alcoholics who are attempting to remain abstinent (Cooney et al. 1997).

Some investigators have attempted to gain the advantages of a realistic drinking setting in the controlled laboratory environment by recreating a barroom in the laboratory, complete with bartender (Marlatt and Gordon 1985; Laberg 1990). The alcoholic beverage is mixed in front of the subject, and he or she is free to pick up the drink, sniff it and, in some circumstances, drink it. The intention is to decrease the inhibiting effect seen in the laboratory and to add as many relevant stimulus cues for drinking as possible, including the sight and smell of alcoholic beverages.

Laberg (1990) has reported significant rates of craving in simulated barroom settings, but these rates have been obtained by allowing alcohol consumption, often in the form of a priming dose. A few studies have been conducted using simulated barrooms with alcoholics in which alcohol consumption has not been allowed.

In one effort to examine elements that contribute to the stimulus value of a setting that might further induce craving, Sher (1985) arranged for social drinkers (i.e., those who consume at least two drinks per week) to be served either a nonalcoholic drink or an alcoholic drink either alone or in a group setting in a room designed to resemble a lounge with a bar and bartender. The presence of a group of drinkers appeared to interact with the barlike setting to increase the positive mood and the perceived pleasurable physical sensations in response to the beverage. Indeed, the subjects who consumed a neutral beverage in a group setting reported physical sensations similar to those reported by subjects who had consumed alcohol. The implication of this study for laboratory craving research is that approximating the setting in which the subject usually drinks, including the social context (e.g., a group setting), may be useful in enhancing the craving response. However, a reading of the existing studies suggests that without the subject having some expectation of being able to consume alcohol, these manipulations may not be sufficient to reliably elicit craving.

## Conclusions

The idea that a cue-exposure procedure in the laboratory can be used to study craving is so appealing that dozens of investigators have produced studies on the topic. Theoretically, it would seem simple to construct a stimulus that would elicit craving, even in a controlled setting like a laboratory. Yet, for the most part, success in inducing craving in the laboratory has been inconsistent. Although most studies report that aggregate desire for alcohol increases with exposure to various stimuli, few studies report the percentage of subjects that experience an increase in craving. Those studies that include percentages typically report that only about 50 to 66 percent of the subjects experience an increase in craving. The induction procedures that have proven most successful thus far are those that employ guided imagery to expose the subject to as many alcohol-related cues as possible, both external and internal, while suggesting, through the use of carefully worded instructions, some possibility that the person may drink if absolutely necessary (Cooney et al. 1997; Rohsenow et al. 1994).

Although guided imagery techniques have shown some promise in eliciting craving in subjects, they are nevertheless problematic. The relaxation that inevitably takes place in such procedures may act to dampen craving reactivity (Litt et al. 1990) while the imagery simultaneously encourages reactivity. Furthermore, the nature of the guided imagery task is such that experimenter demand for reporting craving is maximized. That is, in some guided imagery studies, subjects are explicitly instructed to experience craving and are then asked to report if they really experience craving or not. Given these instructions, participants may feel obliged to report craving even if they do not feel it. Finally, few studies have sought to validate the cue reactivity found in the laboratory by linking reactivity to actual drinking behavior.

For the craving-induction procedure to function as a laboratory model of high-risk situations outside of the laboratory setting, a connection should exist between reactivity elicited in the laboratory and subsequent drinking behavior. Some evidence for this connection has been found using guided imagery inductions (see Cooney et al. 1997), but the results overall are mixed, with several studies reporting no relation between reactivity in the laboratory and drinking after treatment. To conduct valid craving studies, investigators must demonstrate more than just self-reported or physiological reactivity in the laboratory.

Setting manipulations, such as simulating barrooms, complete with bartenders and fellow drinkers, and preferably located away from the usual laboratory environment, may provide a sufficiently stimulating environment to overcome the artificiality of the situation. In the meantime, guided imagery inductions remain the most efficient means for trying to induce craving without encouraging drinking. It may be the case, however, that no laboratory model of craving will prove adequate to capture the phenomenon that alcoholics say they experience. Ultimately, researchers may need to study alcoholics within their home environments and monitor their perceptions in order to provide the basis for a universally accepted definition of craving.

## Footnotes

1 Symptoms of acute alcohol withdrawal occur within hours of terminating a prolonged drinking bout and may include sweating, tremor, rapid heart beat, flushing, and convulsions.

2 This “mixing ritual” is analogous to the “cook-up ritual” used by Childress and colleagues (1993) to study cue reactivity among opiate abusers.

3 Social drinkers in this study consumed an average of three to four drinks per week.

4 “Potency” as defined in this study has no relation to a beverage’s alcohol content. The substance used to provide the low-potency neutral odor was soap.

5 The alcoholic beverage used in the drinking test in all cases was vodka.
